# A risk prediction model for neovascular glaucoma secondary to proliferative diabetic retinopathy based on Boruta feature selection and random forest

**DOI:** 10.3389/fcell.2025.1604832

**Published:** 2025-06-27

**Authors:** Zihan Huang, Di Gong, Cuicui Tang, Jinghui Wang, Chenchen Zhang, Kuanrong Dang, Xiaoyan Chai, Jiantao Wang, Zhichao Yan

**Affiliations:** ^1^ The Second Clinical Medical College of Jinan University, Shenzhen Eye Hospital, Shenzhen, Guangdong, China; ^2^ Shenzhen Eye Hospital, Shenzhen Eye Institute, Southern Medical University, Shenzhen, Guangdong, China; ^3^ General Hospital of Southern Theater Command of PLA, Ophthalmology Department, Guangzhou, Guangdong, China; ^4^ The First Affiliated Hospital of Jinan University, Jinan University, Guangzhou, Guangdong, China

**Keywords:** diabetic retinopathy, random forest, Boruta feature selection, neovascular glaucoma, risk prediction model

## Abstract

**Background:**

Neovascular glaucoma (NVG) is one of the most severe complications of proliferative diabetic retinopathy (PDR), carrying a high risk of blindness. Establishing an effective risk prediction model can assist clinicians in early identification of high-risk patients and implementing personalized interventions to reduce the incidence of vision impairment. This study aimed to develop and evaluate a risk prediction model for NVG in PDR patients based on the Boruta feature selection method and random forest algorithm to improve clinical predictive performance.

**Methods:**

This retrospective study included 365 PDR patients treated at Shenzhen Eye Hospital between January 2019 and December 2024, comprising 269 controls (non-NVG) and 96 cases (NVG). The Boruta feature selection method was employed to identify key features associated with NVG development in PDR. A risk prediction model was then constructed using the random forest algorithm. Model performance was evaluated based on accuracy, sensitivity, specificity, and area under the curve (AUC). Additionally, calibration curves and decision curve analysis (DCA) were used to assess clinical utility. All data analyses and modeling were performed in R (version 4.2.3).

**Results:**

The Boruta algorithm selected 12 significant predictive features. The random forest-based model achieved an accuracy of 90.74%, sensitivity of 82.14%, specificity of 93.75%, and an AUC of 0.87, demonstrating strong predictive performance. Calibration curves indicated reliable prediction probabilities within the 0.4–0.8 range. Decision curve analysis revealed substantial clinical net benefit across threshold probabilities of 0.2–0.8.

**Conclusion:**

The Boruta-guided random forest model developed in this study exhibits excellent predictive performance and clinical applicability for assessing NVG risk in PDR patients.

## 1 Introduction

Diabetes mellitus (DM) is a globally prevalent metabolic disorder characterized by chronic hyperglycemia, which can lead to various chronic complications, including cardiovascular disease, nephropathy, neuropathy, and retinopathy (Kharroubi and Darwish, 2015). Diabetic retinopathy (DR), one of the most common microvascular complications of diabetes, is also a leading cause of preventable blindness in adults worldwide. Epidemiological studies indicate that approximately 30%–40% of diabetic patients develop DR (Yau et al., 2012; Ruta et al., 2013). The risk of DR increases with the duration of diabetes, and poor glycemic control, hypertension, and dyslipidemia can accelerate its progression (Yau et al., 2012; Cheung et al., 2010; Lin et al., 2021). It is estimated that about one-third of diabetic patients suffer from DR, with some progressing to severe retinopathy (Yau et al., 2012; Cheung et al., 2010; Klein et al., 2008; Teo et al., 2021). Furthermore, epidemiological projections suggest that the global burden of DR is not only increasing but also shifting from high-income countries to middle-income regions, which may lead to a rise in other ocular complications associated with DR (Tan and Wong, 2022).

DR can be classified based on disease severity into non-proliferative diabetic retinopathy (NPDR) and proliferative diabetic retinopathy (PDR) (Yeh et al., 2003; Yang et al., 2022). NPDR is primarily characterized by increased retinal capillary permeability, leading to manifestations such as microaneurysms and hard exudates. In contrast, PDR results from retinal ischemia and hypoxia, stimulating neovascularization, which increases the risk of vitreous hemorrhage, tractional retinal detachment, and macular edema (Cheung et al., 2010; Kollias and Ulbig, 2010; Simo-Servat et al., 2019). Additionally, abnormal neovascularization may extend to the anterior chamber angle, obstructing aqueous humor outflow and potentially leading to neovascular glaucoma (NVG) (Tang et al., 2023; Senthil et al., 2021; Calu et al., 2022).

NVG is one of the severe late-stage complications of DR, arising from retinal ischemia-induced abnormal expression of pro-angiogenic factors such as vascular endothelial growth factor (VEGF). This leads to pathological neovascularization of the iris and anterior chamber angle, ultimately causing angle closure and refractory intraocular hypertension (Tang et al., 2023; Senthil et al., 2021). NVG is characterized by insidious onset, rapid progression, difficulty in controlling intraocular pressure, and a high blindness rate. Without timely intervention, it can result in irreversible optic nerve damage and eventual vision loss. According to reports, within 5 years of initial diagnosis of type 2 diabetes, 1.74% (1,249 out of 71,817 patients) developed PDR, 0.25% developed tractional retinal detachment (TRD), and 0.14% developed NVG (Gange et al., 2021). Therefore, early identification of NVG risk in PDR patients and effective intervention are crucial for improving visual prognosis and reducing the risk of blindness.

Currently, the clinical prediction of NVG primarily relies on ophthalmologists’ empirical judgment and certain clinical risk factors, such as severe PDR, vitreous hemorrhage, retinal vein occlusion, prolonged diabetes duration, and poor glycemic control. However, traditional methods often struggle to accurately quantify individualized risk and fail to fully account for the complex interactions among multiple factors, resulting in limited predictive accuracy.

In recent years, with advancements in artificial intelligence and machine learning technologies, machine learning models have been increasingly applied in the medical field. These models demonstrate superior performance, particularly in disease risk prediction, diagnosis, and personalized treatment decision-making. In the field of ophthalmology, following Yang Weihua et al.’s proposal of “Intelligent Ophthalmology” (IO) has flourished remarkably in this domain. IO aims to utilize advanced smart technologies to enhance comprehensive management of all aspects of eye health throughout the entire life cycle. This approach is designed to provide patients with superior healthcare experiences and enhanced health protection (Gong et al., 2024).

In the current era of proliferating modeling approaches, while logistic regression models maintain advantages including structural simplicity, strong interpretability, and the ability to provide explicit coefficient-based explanations for variable effects, their reliance on linear assumptions and feature engineering fundamentally limits their performance when handling data with complex nonlinear relationships. Neural network models demonstrate powerful fitting capabilities when processing high-dimensional data (e.g., images or text), yet they demand substantial data volumes and computational resources. Their black-box nature also limits applications in domains requiring explicit interpretation, such as healthcare. In contrast, the Cox proportional hazards model offers unique advantages in survival analysis by effectively handling time-to-event data, though it relies on the proportional hazards assumption and exhibits weaker adaptability to nonlinear relationships.

Among these methods, Random Forest (RF)—an ensemble learning approach based on decision trees—has significantly reduced preprocessing burdens in modeling due to its notable advantages including strong nonlinear modeling capacity, robust resistance to overfitting, and capability to handle high-dimensional data (Degenhardt et al., 2019; Jin, 2024). Furthermore, its ensemble mechanism effectively mitigates overfitting risks through voting or averaging across multiple decision trees, thereby enhancing model generalizability. These characteristics have led to its widespread application in medical prediction modeling (Hu and Szymczak, 2023; Gelbard et al., 2023; Lilhore et al., 2023; Shi et al., 2023).

Meanwhile, in practical modeling processes, the selection of feature variables is crucial for the predictive performance of the model. High-dimensional data may contain numerous redundant or irrelevant variables and directly inputting all variables can lead to increased model complexity, higher computational costs, and even reduced generalization ability. Therefore, efficient feature selection methods are essential for improving both the performance and interpretability of predictive models. Boruta, a feature selection algorithm based on random forests, is named after the Slavic Forest deity and was developed to identify all relevant variables within a classification framework (Kursa and Rudnicki, 2010). By introducing “shadow features” and performing multiple rounds of random forest computations, it effectively identifies significant variables while excluding irrelevant or redundant ones. In each iteration, the predictor set is doubled by adding a shuffled copy of each original variable. These shadow features are generated by permuting the original values across observations, thereby disrupting their relationship with the outcome. This method has been widely applied in biomedical data analysis, enhancing model stability, reducing dimensionality, and improving interpretability. In contrast, the Boruta feature selection method combined with the Random Forest model achieves a balanced performance across multiple dimensions.

Therefore, this study aims to employ the Boruta feature selection algorithm combined with a random forest model to construct a machine learning-based risk prediction model for NVG in patients with PDR. The objective is to develop a stable, efficient, and highly interpretable NVG prediction model to assist clinicians in earlier identification of high-risk patients, enable personalized management, reduce blindness risk associated with NVG, and improve visual prognosis in diabetic patients.

## 2 Materials and methods

### 2.1 Study design

**FIGURE 1:**
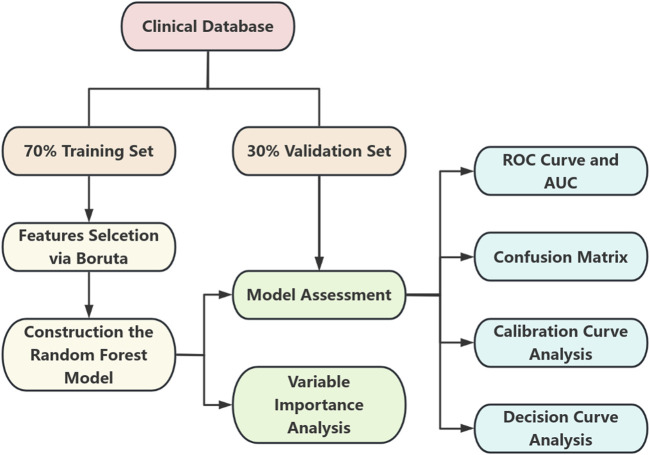
Flowchart depicting a clinical database split into a seventy percent training set and thirty percent validation set. The training set undergoes feature selection via Boruta, leading to the construction of a random forest model. Both sets contribute to model assessment. The outcomes include variable importance analysis, ROC curve and AUC, confusion matrix, calibration curve analysis, and decision curve analysis.

This study adopted a retrospective design, enrolling a total of 365 PDR patients treated at Shenzhen Eye Hospital between January 2019 and December 2024. Based on NVG comorbidity status, patients were divided into two groups: the control group (non-NVG patients, n = 269) and the case group (NVG patients, n = 96). The study aims to identify clinically significant feature variables closely associated with NVG development by analyzing clinical data, medical history, and metabolic-related parameters, ultimately constructing a machine learning-based risk prediction model for NVG occurrence in PDR patients. The overall workflow of this study is illustrated in Figure 1.

The detailed sample selection criteria were as follows: Inclusion criteria, 1) male and female patients aged 18 years or older, 2) patients clinically diagnosed with diabetic retinopathy (including all DR types) within the past 3 years, 3) patients with detailed disease course records and complete clinical data including biomarker profiles. Exclusion criteria, 1) presence of severe congenital eye diseases or other ocular pathologies that could interfere with assessment, such as severe dry eye syndrome or corneal disorders, 2) patients with insufficient clinical data or incomplete medical records, 3) patients suffering from severe systemic diseases including end-stage renal disease or cardiac conditions.

This study strictly adhered to the principles of the Declaration of Helsinki and relevant ethical requirements. As it did not involve direct patient intervention or treatment, all patient privacy and data security were ensured. The research protocol was approved by the Ethics Committee of Shenzhen Eye Hospital (Ethics Approval No. 2025KYPJ008).

### 2.2 Data collection

This study retrospectively collected multiple clinical data from PDR patients, including basic patient information, medical history, ophthalmic surgical history, and relevant metabolic and biochemical indicators. The basic information included patient name, hospitalization number, age, gender, eye laterality (right/left eye), and best-corrected visual acuity (BCVA). Regarding medical history, data on hypertension history, diabetes duration, coronary heart disease history, diabetic nephropathy history, and stroke history were collected. Ophthalmic surgical history included records of intravitreal anti-VEGF drug therapy, retinal laser photocoagulation surgery, and pars plana vitrectomy (PPV) surgery. Metabolic and biochemical indicators involved body mass index (BMI), blood glucose (GLU), urinary glucose (UG), urinary protein (UP), Alanine Aminotransferase (ALT) and Aspartate Aminotransferase (AST), and serum creatinine (CREA) and uric acid (UA). For the collected categorical variables (e.g., gender, eye laterality, and medical history), specific numerical coding was applied, with detailed variable assignment schemes presented in Table 1.

**TABLE 1:** Detailed variable assignment schemes.

Full Variable Name	Variable Abbreviation	Assignment Description
Outcome	Outcome	0 = non-NVG; 1 = NVG
Name	Name	Text variable
Hospitalization ID	ID	Text variable
Age	Age	Numerical variable
Gender	Gender	1 = Male; 2 = Female
Eye Laterality	Laterality	1 = Right eye; 2 = Left eye
LogMAR Visual Acuity	BCVA	Numerical variable
Systolic Blood Pressure	SBP	Numerical variable
Diastolic Blood Pressure	DBP	Numerical variable
Hypertension History	HBP	0 = No; 1 = Yes
Diabetes Duration	Diabetes Duration	1 = ≤5 years; 2 = 5–10 years; 3 = 10–15 years; 4 = ≥15 years
Coronary Heart Disease History	CHD	0 = No; 1 = Yes
Diabetic Nephropathy History	DN	0 = No; 1 = Yes
Stroke History	Stroke	0 = No; 1 = Yes
Diabetes-Related Amputation History	Amputation	0 = No; 1 = Yes
Intravitreal Anti-VEGF Therapy History	Anti-VEGF	0 = No; 1 = Yes
Family History of Glaucoma	Family History	0 = No; 1 = Yes
Retinal Laser Photocoagulation History	RLP	0 = No; 1 = Yes
Pars Plana Vitrectomy History	PPV	0 = No; 1 = Yes
Lens Removal Surgery History	Lens Removal	0 = No; 1 = Yes
Intraocular Lens Implantation History	IOL	0 = No; 1 = Yes
Body Mass Index	BMI	Numerical variable
Alanine Aminotransferase	ALT	Numerical variable
Aspartate Aminotransferase	AST	Numerical variable
Blood Urea Nitrogen	BUN	Numerical variable
Serum Creatinine	CREA	Numerical variable
Uric Acid	UA	Numerical variable
Blood Glucose	GLU	Numerical variable
Urinary Glucose	UG	0 = Negative; 1 = Positive or Suspiciously Positive
Urinary Protein	UP	0 = Negative; 1 = Positive or Suspiciously Positive

### 2.3 Data processing

Missing values were imputed using the Predictive Mean Matching (PMM) method to minimize data bias and enhance model stability. After handling missing data, the dataset was randomly split into training and validation sets at a 7:3 ratio. The training set was used for feature selection and model development, while the validation set was reserved for model evaluation.

### 2.4 Boruta feature selection

The Boruta algorithm was implemented in the training set using NVG status (Outcome) as the dependent variable and all other variables as independent variables. Through the creation of randomized shadow features and iterative computation of variable importance, Boruta identified statistically significant predictors influencing NVG development risk. These selected features were subsequently used for model construction.

### 2.5 Random forest modeling and evaluation

RF algorithm was employed as the core modeling approach, with cross-validation used for hyperparameter optimization to enhance model generalizability. Model performance was then evaluated on the validation set using metrics including the area under the curve (AUC), sensitivity, specificity, and F1-score. A confusion matrix was generated to visualize the classification results, demonstrating the alignment between predicted and true outcomes. Furthermore, a calibration curve was utilized to assess the accuracy of predicted probabilities, while decision curve analysis (DCA) was performed to evaluate the model’s clinical utility, thereby validating its practical applicability in clinical settings.

### 2.6 Statistical software

All data analysis and modeling procedures were conducted using R language (version 4.2.3). The following R packages were specifically employed: The Boruta package for random forest-based feature selection. The random Forest package for model construction and hyperparameter tuning. The pROC package for ROC curve analysis and AUC calculation. The caret package for cross-validation and model evaluation. The mice package implementing PMM for missing value imputation. The ggplot2 package for generating calibration curves, decision curves, and confusion matrix visualizations. The complete analytical workflow was executed within the R environment to ensure scientific rigor and result accuracy.

## 3 Result

### 3.1 Baseline characteristics

The unit of analysis in this study was the individual eye (rather than the patient), with each eligible eye independently included for statistical analysis. Based on the predefined inclusion and exclusion criteria, the final cohort comprised 258 patients (365 qualifying eyes). The specific group distribution was as follows: the DR group included 179 patients (269 eyes total), while the NVG group consisted of 79 patients (96 eyes total).

In terms of age, patients in the DR group were 58.56 ± 10.34 years old while NVG patients were 58.39 ± 13.08 years old, indicating an elderly patient population overall. For gender distribution, the DR group showed significant disparity between male patients (195 eyes) and female patients (74 eyes), demonstrating male predominance. Similarly, in the NVG group, male patients (71 eyes) substantially outnumbered female patients (25 eyes), also exhibiting male dominance. The distribution between right and left eyes was relatively balanced across all groups. The baseline characteristics of each group are described in Table 2.

**TABLE 2:** Baseline characteristics of study groups.

Parameter	DR Group	NVG Group
Number of patients	179	79
Age (years)	58.56 ± 10.34	58.39 ± 13.08
Gender (Male/Female)	133/46	58/21
Sample size (eyes)	269	96
Laterality (Right/Left)	142/127	46/50

### 3.2 Boruta feature selection results

The Boruta algorithm identified the following variables as having significant predictive value: Age, BCVA, Diabetes Duration, RLP, PPV, Lens Removal, IOL, BMI, ALT, BUN, CREA, and UA. These key features demonstrate important clinical predictive value for DR patients and were subsequently used for risk prediction model construction and optimization.

### 3.3 Model performance comparison and algorithm selection

**FIGURE 2:**
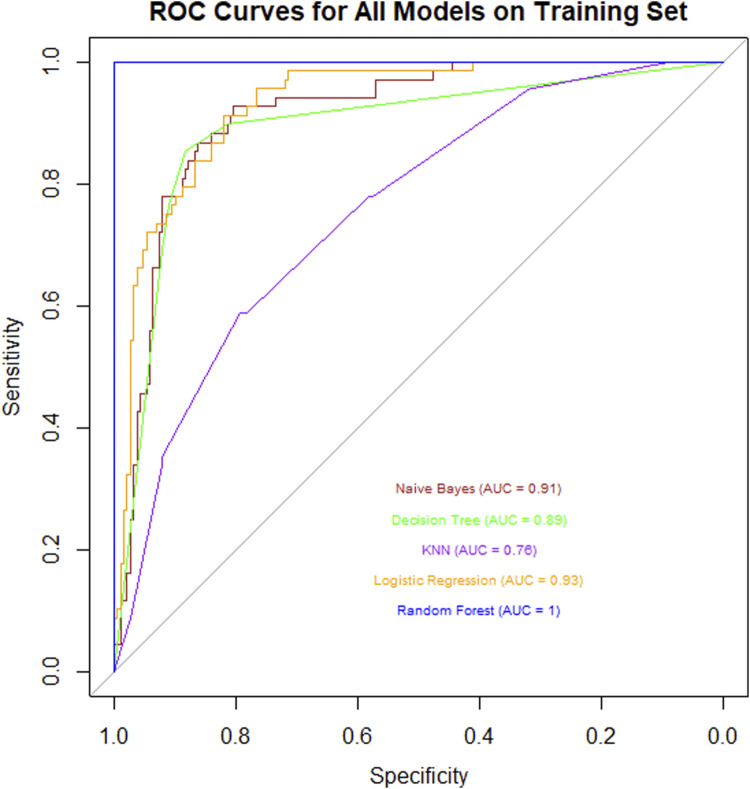
ROC curve chart for various models on a training set, showing Naive Bayes (AUC = 0.91), Decision Tree (AUC = 0.89), KNN (AUC = 0.78), Logistic Regression (AUC = 0.93), and Random Forest (AUC = 1). Sensitivity is plotted against 1-Specificity. Random Forest shows the highest performance.

In this study, we conducted a systematic analysis of the training dataset using multiple classical machine learning algorithms to evaluate their performance in the target classification task. Specifically, we compared the classification efficacy of Naïve Bayes, Decision Tree, K-Nearest Neighbors (KNN), Logistic Regression, and Random Forest, with quantitative assessment based on the Area Under the Curve (AUC) of the Receiver Operating Characteristic (Figure 2).

The experimental results revealed significant differences in AUC values across the algorithms: Naïve Bayes (AUC = 0.91) demonstrated strong probabilistic modeling capabilities, while Decision Tree (AUC = 0.89) exhibited robust feature partitioning performance. In contrast, KNN (AUC = 0.76) showed limited performance, potentially due to sensitivity to data dimensionality or noise. Logistic Regression (AUC = 0.93) achieved excellent results owing to its linear separability advantages. Remarkably, Random Forest (AUC = 1.00) attained perfect classification through ensemble learning mechanisms (Bootstrap Aggregating and random subspace feature selection), with its generalization capability and anti-overfitting properties significantly outperforming other models.

This comprehensive comparison highlights Random Forest as the optimal choice for the given classification task, supported by its superior predictive accuracy and robustness.

### 3.4 Random forest model performance

Based on the Boruta feature selection results, this study successfully constructed a risk prediction model for NVG development in PDR patients using the random forest algorithm. To comprehensively evaluate model performance, we calculated the ROC curve and AUC value on an independent validation set, supplemented by confusion matrix analysis, calibration curve assessment, and DCA. Furthermore, variable importance analysis was conducted to interpret the model’s decision-making logic.

#### 3.4.1 ROC curve and AUC

**FIGURE 3:**
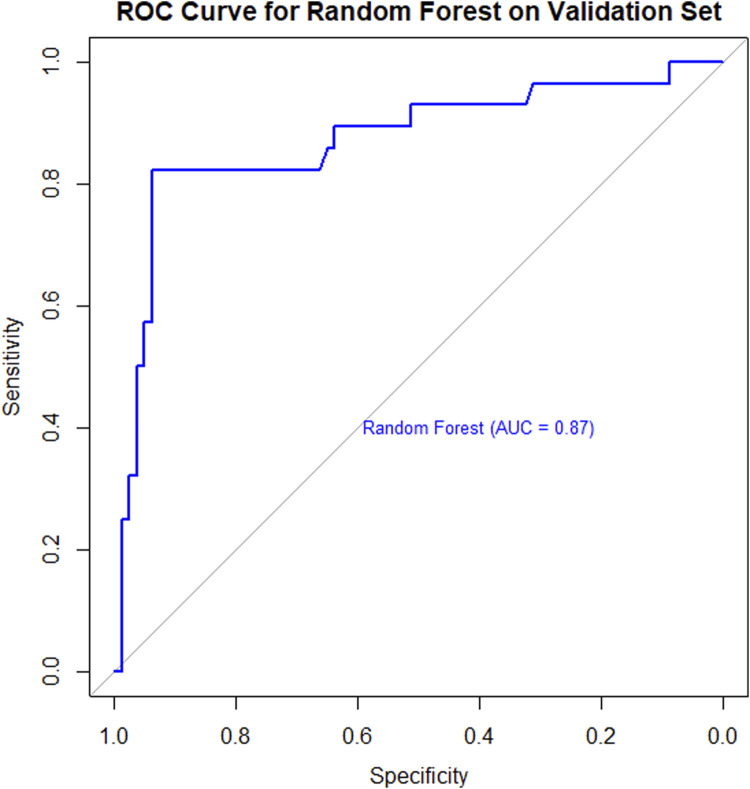
ROC curve for a Random Forest model on a validation set. The curve shows a blue line with an Area Under the Curve (AUC) of 0.87, indicating strong model performance. Sensitivity is plotted on the y-axis and specificity on the x-axis.

The model’s discriminative ability was evaluated on the validation set. Results demonstrated that the random forest model exhibited strong discriminatory performance, with an AUC value of 0.87, indicating high predictive accuracy for identifying NVG risk in PDR patients (Figure 3).

#### 3.4.2 Predictive performance and confusion matrix

**FIGURE 4:**
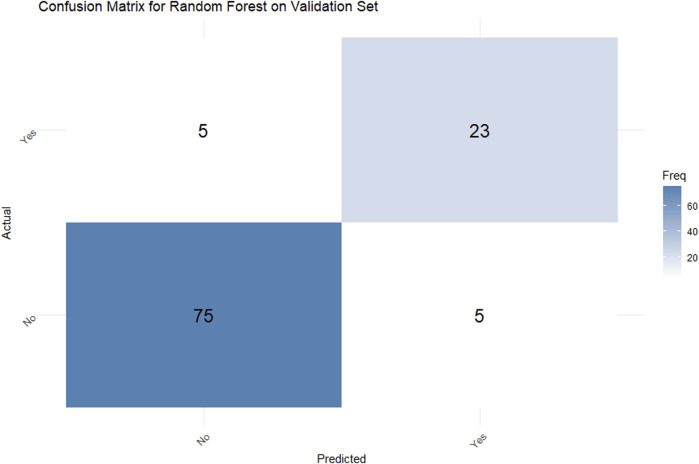
Confusion matrix for a Random Forest model on a validation set. The actual versus predicted comparison shows: True Positives: 5, False Positives: 23, False Negatives: 75, and True Negatives: 5. A blue color gradient represents frequency, with darker shades indicating higher values.

In the validation set, the random forest model achieved a classification accuracy of 90.74% (95% CI: 83.63%–95.47%). The confusion matrix (Figure 4) demonstrated a sensitivity of 82.14% and specificity of 93.75%, indicating robust performance in discriminating between PDR patients with and without NVG. Additionally, the Kappa coefficient of 0.7589 confirmed strong agreement between model predictions and true classifications, significantly reducing the influence of random chance.

#### 3.4.3 Calibration curve analysis

**FIGURE 5:**
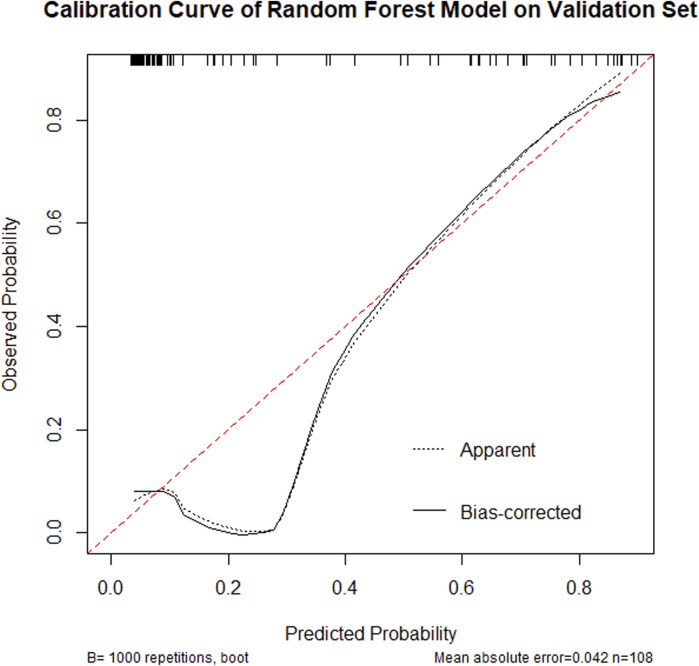
Calibration curve of a Random Forest model on a validation set, showing observed probability versus predicted probability. The dashed line indicates apparent predictions, while the solid line represents bias-corrected predictions. The curve shows good predictive alignment with a mean absolute error of 0.042, based on 108 samples, repeated 1,000 times.

The calibration curve (Figure 5) demonstrated that the model achieved a mean absolute error of 0.042 and mean squared error of 0.00484 between predicted and observed probabilities, indicating high predictive accuracy across different probability thresholds. Particularly within the 0.4–0.8 probability range, the calibration curve closely approximated the ideal reference line (45° diagonal), confirming excellent calibration performance in this critical clinical decision-making range.

#### 3.4.4 Decision curve analysis

**FIGURE 6:**
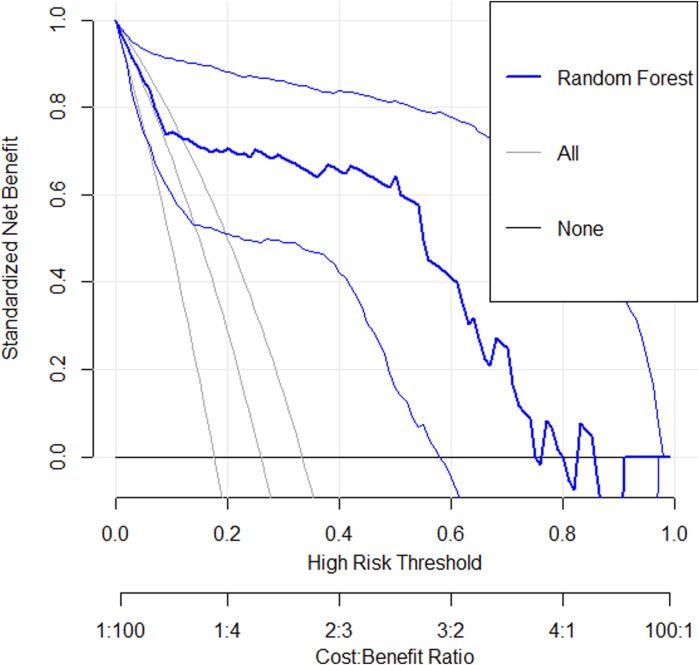
A decision curve analysis graph shows standardized net benefit versus high-risk threshold. Three lines are depicted: “Random Forest” in bold blue, “All” in light gray, and “None” in black. The x-axis represents high-risk thresholds from 0 to 1, with a cost-benefit ratio beneath. The y-axis indicates the standardized net benefit from 0 to 1. The Random Forest model shows better performance across most thresholds compared to All and None.

The DCA (Figure 6) showed that across the 0.2–0.8 decision threshold range, the random forest model’s net benefit consistently exceeded both the treat-all and treat-none baseline strategies, demonstrating superior clinical decision-making utility within this threshold range.

#### 3.4.5 Variable importance analysis

**FIGURE 7:**
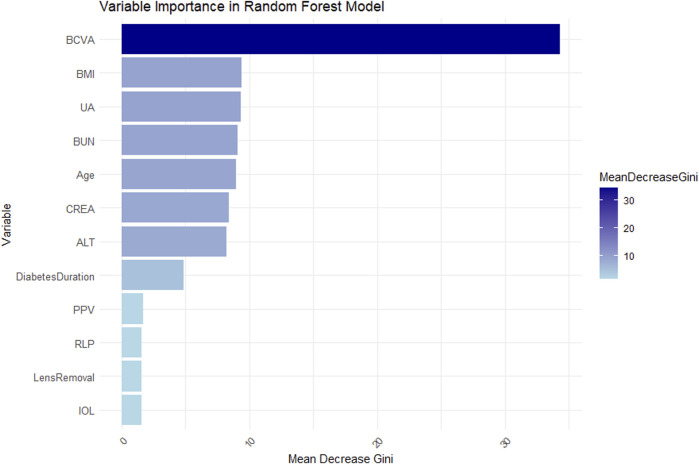
Bar chart showing variable importance in a Random Forest model. BCVA has the highest mean decrease Gini score, followed by BMI, UA, BUN, Age, CREA, and ALT. Diabetes Duration, PPV, RLP, Lens Removal, and IOL have lower scores. A color gradient indicates the mean decrease in Gini, ranging from light to dark blue.

To enhance model interpretability, this study calculated feature importance based on Gini index. The variables were ranked by their mean decrease in Gini index (Figure 7). Results showed BCVA, BMI, UA, BUN, Age, CRE, ALT, and Diabetes Duration were key decision-making variables, likely playing important roles in disease prediction and clinical assessment. In contrast, PPV, RLP, Lens Removal and IOL showed relatively lower contributions, having limited impact on predictions in the current model.

## 4 Discussion

### 4.1 Main findings

This study aimed to develop a risk prediction model for NVG in PDR patients using Boruta feature selection and random forest algorithms. Through retrospective analysis of clinical data from PDR patients combined with Boruta feature selection, we successfully identified multiple clinically relevant variables significantly associated with NVG development. Twelve key factors were found to substantially influence NVG occurrence: Age, BCVA, Diabetes Duration, RLP, PPV, Lens Removal, IOL, BMI, ALT, BUN, CREA, and UA.

The RF model, serving as the core predictive tool in this study, demonstrated superior predictive performance on the test dataset with an accuracy of 90.74%, sensitivity of 82.14%, specificity of 93.75%, and AUC of 0.87. These metrics indicate its excellent discriminative ability and accuracy in predicting NVG risk among PDR patients. Furthermore, calibration curve analysis revealed high consistency between predicted probabilities and actual observations across various probability thresholds, confirming the model’s reliability at different risk levels. Decision curve analysis showed that within the 0.2–0.8 decision threshold range, the random forest model provided significantly higher clinical net benefit, demonstrating not only outstanding statistical performance but also superior predictive utility for clinical decision-making. In summary, our risk prediction model exhibits strong potential for clinical application, offering an effective tool for early screening of NVG and personalized treatment strategies in diabetic patients.

### 4.2 Advantages and limitations compared to previous studies

DR, as one of the most prevalent microvascular complications of diabetes, demonstrates a continuously rising global prevalence, particularly among patients with poor glycemic control and prolonged disease duration. Epidemiological studies indicate that the incidence of DR exhibits an upward trend parallel to the increasing prevalence of diabetes, with PDR patients facing substantially higher risks of vision loss (Kour et al., 2024). Current research on DR-induced NVG primarily focuses on three key aspects: epidemiological characteristics, pathogenic mechanisms, and advanced therapeutic strategies (Lin et al., 2021; Liu and Wu, 2021). NVG, as a severe complication of PDR, is characterized by high blindness rates and challenging treatment, making early identification of high-risk patients crucial. The development of NVG is closely associated with VEGF overexpression secondary to retinal ischemia. Current treatment strategies include anti-VEGF agents, panretinal photocoagulation, and glaucoma surgeries. However, therapeutic outcomes vary significantly, with some patients still experiencing irreversible optic nerve damage due to angle closure and refractory intraocular pressure elevation despite standardized treatment. While existing studies predominantly focus on comparing NVG treatment modalities (Lin et al., 2025; Lin et al., 2022), such as anti-VEGF combined with trabeculectomy or analysis of efficacy for intravitreal anti-VEGF combined with Ahmed glaucoma valve implantation, few have addressed early prediction of NVG onset.

This study identified significant associations between NVG risk and clinical characteristics of PDR patients, including BCVA and hepatic/renal function indicators, providing clinicians with more comprehensive risk assessment criteria. These findings facilitate targeted interventions prior to NVG onset and enable early-stage risk stratification, thereby addressing limitations of conventional screening methods. Future work will compare different intervention approaches to determine the optimal strategy for minimizing complication risks, ultimately generating evidence-based clinical recommendations. Compared with previous studies that primarily focused on the diagnosis and treatment of single diseases, analysis of influencing factors for individual diseases (Gong et al., 2023), or explored the potential of AI in disease assessment (Jiang et al., 2024), this study specifically addresses the risk prediction of complications, which holds significant clinical implications.

### 4.3 Analysis of significant features

This study identified 12 key features (including age, BCVA, diabetes duration, BMI, etc.) through Boruta algorithm screening. Below we briefly analyze the potential relationships between these indicators and disease pathogenesis:

BCVA emerged as the most predictive variable in our model. Poor BCVA typically indicates severe retinal pathology, including but not limited to macular edema, vitreous hemorrhage, or tractional retinal detachment. Although visual deterioration may serve as an early warning sign for NVG, clinical observations revealed two high-risk phenomena: first, PDR patients often fail to perceive secondary pathological changes due to gradual vision decline; second, low vision status reduces follow-up compliance, consequently leading to treatment delays.

BMI serves as a simple and rapid clinical indicator for assessing health status. Among different types of diabetic patients, BMI levels may vary significantly. In the diabetic population, elevated BMI (obesity) is closely associated with insulin resistance - a well-established risk factor for DR progression. Furthermore, patients with high BMI frequently develop leptin resistance, and chronically elevated leptin levels may induce endothelial dysfunction, thereby exacerbating microvascular disease risk (Wu et al., 2023).

Hyperuricemia is associated with oxidative stress and endothelial damage (Gherghina et al., 2022), potentially directly stimulating VEGF expression. UA can also activate inflammatory pathways (e.g., the NLRP3 inflammasome) (Wan et al., 2016), and activation of these inflammatory bodies may exacerbate the progression of retinal complications (McCurry et al., 2024). However, whether UA acts as an independent risk factor or merely reflects the overall state of metabolic dysregulation requires further validation.

Blood Urea Nitrogen, as one of the end products of protein metabolism, is primarily synthesized in the liver (via the urea cycle) and excreted by the kidneys. Its serum levels reflect both renal excretory function and protein metabolic status. Elevated BUN indicates impaired renal function, and patients with diabetic nephropathy often present with more severe DR. Renal dysfunction may lead to the accumulation of uremic toxins that damage vascular endothelial function and exacerbate retinal hypoxia through associated anemia (Tonelli et al., 2016). Additionally, DN patients frequently demonstrate poorer blood pressure control, which may further increase NVG risk.

Advanced age demonstrates a significant correlation with the development of diabetic microvascular complications. Elderly PDR patients typically exhibit longer disease duration, and prolonged hyperglycemic states may accelerate retinal ischemia and VEGF overexpression, thereby promoting iris and angle neovascularization. Furthermore, age-related systemic vascular pathologies commonly coexist, exacerbating ocular ischemic-hypoxic conditions and elevating NVG risk. However, age may also influence treatment adherence, as geriatric patients often show suboptimal disease awareness and therapeutic compliance, potentially contributing to disease progression.

CREA serves as a key renal function parameter, with its levels inversely correlating with glomerular filtration rate. Impaired renal function may reduce VEGF clearance, leading to its intraocular accumulation. Furthermore, the uremic milieu promotes oxidative stress and endothelial dysfunction, potentially accelerating PDR progression to NVG (Tonelli et al., 2016). The CREA-NVG association may also involve multiple metabolic pathways.

Elevated ALT levels serve as a sensitive marker for hepatocyte injury. In diabetic patients, increased ALT may indicate non-alcoholic fatty liver disease (NAFLD) - a condition closely associated with microvascular complications. The systemic inflammation in NAFLD patients could exacerbate retinal ischemia through oxidative stress mechanisms (Younossi, 2019; Pouwels et al., 2022). Furthermore, impaired hepatic function may disrupt the clearance or metabolism of pro-angiogenic factors like VEGF, thereby potentiating ocular neovascularization.

Duration of DM represents an independent risk factor for DR progression. Chronic hyperglycemia induces retinal capillary pericyte loss and basement membrane thickening, ultimately leading to ischemic changes. Epidemiological data indicate patients with disease duration exceeding 10 years demonstrate higher susceptibility to DR development (Chamard et al., 2021), with the severity of ischemia directly correlating with NVG risk. Furthermore, long-standing diabetes frequently coincides with other microvascular complications (e.g., nephropathy), which may exacerbate ocular pathology through systemic inflammatory responses.

Vitrectomy is commonly employed for PDR treatment, yet intraoperative manipulations may induce retinal damage, exacerbating ischemia and consequently elevating VEGF production (Wakabayashi et al., 2012; Wakabayashi et al., 2017). Additionally, post-vitrectomy inflammatory responses could promote anterior chamber angle neovascularization (Takayama et al., 2019). Earlier studies reported that cataract surgery might accelerate DR progression (Shah and Chen, 2010), potentially through disruption of the blood-aqueous barrier with subsequent increases in inflammatory and VEGF factor release. Furthermore, aphakia may facilitate greater VEGF diffusion into the anterior chamber. Conversely, intraocular lens implantation might reduce anterior VEGF diffusion, though combined with posterior capsule rupture, it could still elevate NVG risk - suggesting IOL status may represent a confounding factor rather than an independent predictor.

Retinal photocoagulation remains a cornerstone of PDR management. However, inadequate or delayed treatment may perpetuate ischemic conditions, paradoxically promoting NVG. Conversely, extensive photocoagulation could compromise retinal perfusion, exacerbating peripheral ischemia and even inducing anterior segment neovascularization. Thus, the photocoagulation-NVG relationship appears bidirectional, requiring comprehensive evaluation of treatment timing and extent.

### 4.4 Clinical significance of the prediction model

The PDR-related NVG risk prediction model developed in this study, based on Boruta feature selection and random forest algorithm, demonstrates high accuracy, sensitivity, and specificity, enabling clinicians to identify high-risk patients at early disease stages. Early interventions (e.g., anti-VEGF therapy, retinal laser photocoagulation) guided by this model may effectively delay or prevent NVG onset, reduce blindness risk, and improve visual prognosis. By quantifying individualized risk, the model provides scientific evidence to support personalized treatment strategies. For instance, high-risk patients may receive more aggressive interventions, while low-risk patients can avoid overtreatment, thereby optimizing resource allocation.

Hospitals can leverage this model to rationally distribute medical resources by focusing on high-risk populations, improving treatment efficacy and quality. Concurrently, reducing unnecessary follow-ups and examinations for low-risk patients decreases healthcare costs and enhances resource utilization efficiency. Early intervention and personalized management can significantly improve visual outcomes and minimize irreversible optic nerve damage caused by NVG, ultimately enhancing patients’ quality of life while alleviating familial and societal economic burdens.

Furthermore, this model integrates machine learning with clinical data, showcasing artificial intelligence’s potential in medical applications. It establishes a reference paradigm for precision medicine in DR and other ophthalmic diseases, advancing AI implementation in ophthalmology during the big data era.

### 4.5 Limitations and future directions

Although the proposed NVG risk prediction model demonstrates satisfactory performance, several limitations should be acknowledged. First, this study adopted a retrospective design with a relatively limited sample size. Therefore, future research should validate the external validity of this model in multicenter, large-scale cohorts. Second, while the Boruta algorithm and random forest model effectively selected features and established the prediction model, the interpretability remains constrained—particularly with high-dimensional data where the “black-box” nature may hinder widespread clinical adoption. Future research could incorporate advanced interpretability techniques (e.g., SHAP values, LIME) to enhance model transparency and operational utility.

Additionally, although diverse clinical variables were considered, potential factors such as genetic predispositions, environmental influences, and imaging features were not included. Future studies should optimize feature selection by integrating multicenter data, external validation cohorts, and multi-omics approaches (e.g., genomics, metabolomics) to improve predictive accuracy and clinical applicability.

## 5 Conclusion

This study successfully developed a risk prediction model for NVG in PDR patients by integrating the Boruta feature selection algorithm with a random forest model. The model demonstrated excellent performance metrics, achieving 90.74% accuracy, 82.14% sensitivity, 93.75% specificity, and an AUC of 0.87, indicating high predictive precision. Calibration curve analysis confirmed strong predictive consistency within the 0.4–0.8 probability range. Decision curve analysis revealed superior clinical net benefit across the 0.2–0.8 decision threshold spectrum.

The proposed risk prediction model exhibits outstanding accuracy, sensitivity, specificity, and clinical utility, providing an effective tool for early screening and personalized management of NVG in diabetic patients. This finding could hold significant clinical relevance and practical application value for ophthalmologic practice.

## Data Availability

The raw data supporting the conclusions of this article will be made available by the authors, without undue reservation.
